# Infergen Stimulated Macrophages Restrict *Mycobacterium tuberculosis* Growth by Autophagy and Release of Nitric Oxide

**DOI:** 10.1038/srep39492

**Published:** 2016-12-21

**Authors:** Susanta Pahari, Nargis Khan, Mohammad Aqdas, Shikha Negi, Jagdeep Kaur, Javed N. Agrewala

**Affiliations:** 1 Immunology Laboratory, CSIR-Institute of Microbial Technology, Chandigarh, 160036, India; 2 Department of Biotechnology, Panjab University, Chandigarh, 160014, India

## Abstract

IFN alfacon-1 (Infergen) is a synthetic form of Interferon (IFN)-α2b. Infergen has immunomodulatory activity and is effective against *hepatitis C virus*. However, the effect of Infergen (IFG) on *Mycobacterium tuberculosis (Mtb)* has not yet been reported. Therefore, for the first time, we have studied the influence of IFG in constraining the survival of *Mtb* in human macrophages. We observed that IFG significantly enhanced the maturation and activation of macrophages. Further, it substantially augmented the secretion of IL-6, nitric oxide (NO) and antigen uptake. Moreover, macrophages exhibited remarkably higher bactericidal activity, as evidenced by reduction in the *Mtb* growth. Infergen-mediated mechanism was different from the type-1 interferons; since it worked through the activation of NF-κB, phosphorylation of STAT-3 and Akt-PI3K that improved the bactericidal activity through autophagy and NO release. In future, IFG immunotherapy can be a novel strategy for treating patients and controlling TB.

In spite of the fact that effective drugs like rifampicin, isoniazid, ethambutol, pyrazinamide, etc., are available to treat tuberculosis (TB); the disease continues to inflict a detrimental impact on public health worldwide. Nearly one third of the world population is infected with *Mycobacterium tuberculosis (Mtb)* and about 1% develops new infection annually, accounting for total 9.6 million new cases^1^. In 2014, there were about 1.5 million allied deaths; typically occurring in the developing countries [WHO, 2015]. The problem is further aggravated with the emergence of MDR-TB (multi-drug-resistant tuberculosis), XDR (extensively drug-resistant tuberculosis) and TDR (totally drug-resistant tuberculosis) strains of *Mtb*, AIDS-pandemic and failure of BCG vaccine^2^,^3^,^4^. Hence, there is an urgent need to identify newer drugs and develop radical changes in the current therapeutic strategies to control TB.

Interferons (IFNs) are naturally occurring immunomodulatory cytokines. Type-I IFNs are very well-known for their anti-viral activity^5^,^6^,^7^,^8^,^9^. Recently, a non-naturally occurring type-I IFN known as IFN alfacon-1 or Infergen (IFG) has been approved against hepatitis C virus^10^,^11^. Infergen is quite distinct from type-I IFNs, since it is derived from the sequences selected from several α-IFNs (interferon α2a and interferon α2b) and then producing a consensus gene product^12^. The role of type-1 IFNs against *Mtb* has not been clear so far^13^,^14^,^15^,^16^. However like IFN-γ, type-1 IFNs are reported to kill *Mtb* by bolstering host immunity^13^,^14^,^15^,^16^.

Although, IFG shares biological and pharmacological properties with type 1 IFNs, it follows a unique and distinct signaling pathway. Infergen exhibits potent immunomodulatory activity in human cells^17^. Furthermore, it is known to control viral infection by enhancing the activation of T cells through an augmented viral peptide presentation by antigen presenting cells (APCs). Unfortunately, nothing has been reported regarding the therapeutic aspect of IFG on *Mtb*. Therefore, we thought that it would be interesting to study the impact of IFG in restricting the intracellular survival of *Mtb* and the mechanism involved therein.

Autophagy plays an essential role in protection against *Mtb*^18^. It targets the *Mtb* antigens to lysosomes for their degradation and clearance. Further, autophagy enhances the antigen presenting ability of APCs to T cells^19^,^20^. At the same time, it inhibits detrimental inflammatory responses^21^. Nitric oxide (NO) is known to confine the growth of *Mtb*^22^. Taking into account these evidences, we were interested to monitor the influence of IFG on the activation of *Mtb* infected macrophages, thereby restricting the survival of the *Mtb*. It was quite fascinating to observe that IFG stimulated macrophages displayed significantly improved skill to restrict the intracellular growth of *Mtb*. The mechanism involved was through the augmentation of autophagy and release of NO. Both these pathways are considered crucial in curbing the growth of *Mtb*. Therefore, this study illustrates that IFG may have enough potential to work as an immunotherapeutic agent against TB.

## Results

### IFG restricts the intracellular survival of *Mtb*

**Figure 1:**
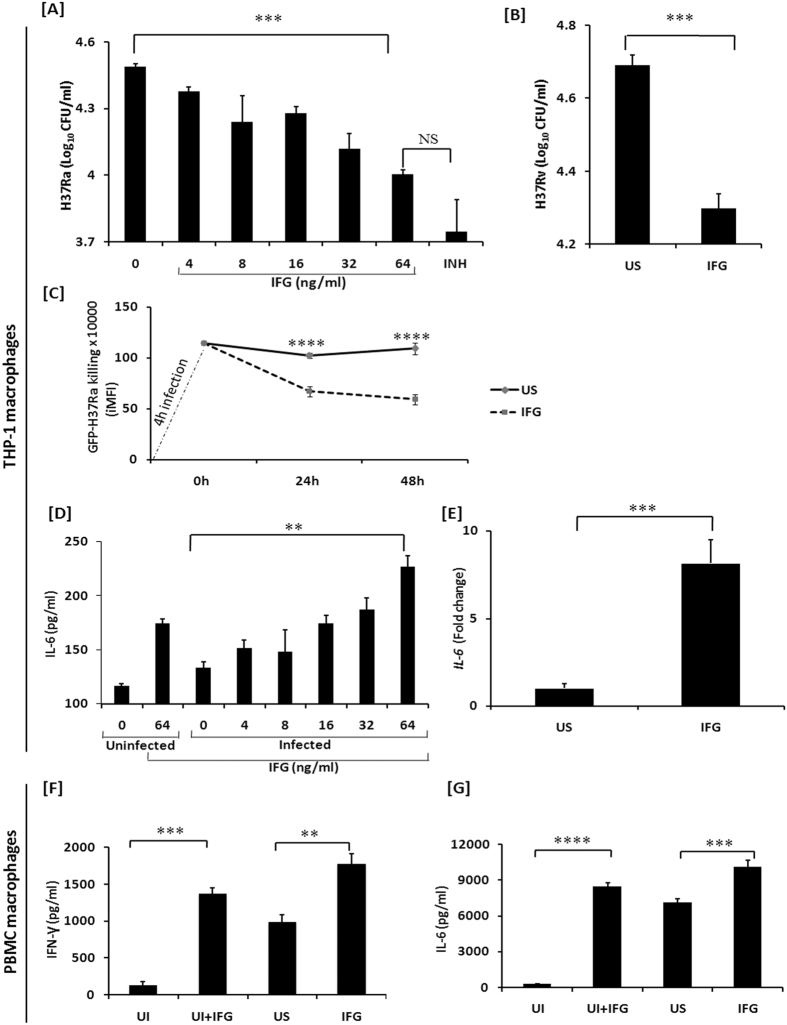
IFG stimulated macrophages show augmented killing of *Mtb.* Macrophages were infected for 4 h with the mycobacterial strains [**A**] H37Ra; [**B**] H37Rv. Later, cells were stimulated with the indicated doses of IFG for 24 h. The infected macrophages were lysed and CFUs were enumerated after 21d. Infected macrophages treated with INH (25 μg/ml) were used as positive control. [**C**] Time-point kinetics of GFP-H37Ra after 4 h infection. This was followed by stimulation with IFG for 24 h and 48 h. The flow cytometry data (iMFI) represented through the line diagrams as mean ± SEM are representative of two independent experiments. [**D**] Supernatants of the H37Ra infected macrophages stimulated with IFG were collected after 24 h for the estimation of IL-6 by ELISA; [**E**] RNA was isolated after 6 h to monitor the expression of *IL-6* by RT-qPCR. Data depicted as bar diagram are mean ± SEM of 4 individual wells and representative of four independent experiments. **p < 0.001, ***p < 0.0001. [**F,G**] Macrophages isolated from human PBMCs were infected with *Mtb* for 4 h. Later, they were stimulated with IFG for 24 h and the supernatants were collected for the estimation of IFN-γ and IL-6. UI: uninfected control; US: *Mtb* infected macrophages; IFG: *Mtb* infected and IFG (64 ng/ml) stimulated macrophages; INH: *Mtb* infected macrophages treated with isoniazid. Data represented as the mean ± SEM are of four wells and two independent experiments. **p ≤ 0.006, ***p ≤ 0.0006, ****p < 0.0001.

*Mtb* not only infects macrophages but also it can survive in the hostile environment of these cells^23^,^24^. Thus, in the initial phase of the study, we investigated the impact of IFG on avirulent strain of *Mtb* H37Ra infected THP-1 macrophages (H37Ra-Mφ). It was quite exciting to note that IFG stimulated macrophages (MφIFG) showed significant (p < 0.0001) reduction in the intracellular survival of *Mycobacterium*, as compared to unstimulated control [Fig. 1A]. The decrease in the CFUs was observed in a dose dependent fashion. Likewise, substantial (p < 0.0008) decline in the growth of virulent strain H37Rv (*Mtb*) was noticed [Fig. 1B]. Furthermore, we proved the above event through time-point kinetics by demonstrating a extensive (p < 0.0001) increase in *Mtb* killing (GFP-H37Ra) by flow cytometry assay [Fig. 1C]. Thus, validating the potent role of IFG in inhibiting the growth of both virulent and avirulent *Mtb*. The concentration of 64 ng/ml of IFG showed the maximum intracellular killing of *Mtb* and was not toxic to Mφ and human peripheral blood mononuclear cells (PBMCs) [Fig. 1A and Fig. S1A–C]. Hence, this dose was chosen in all the subsequent experiments.

### IFG augments the secretion of cytokines

IL-6 is a major cytokine produced by macrophages in response to intracellular pathogens^25^. This cytokine plays an important role in T cell activation and inhibition of the growth of *Mtb*^25^,^26^,^27^,^28^,^29^,^30^,^31^. IFG stimulated H37Ra infected Mφ produced considerably (p < 0.001) higher level of IL-6, as compared to unstimulated (no Infergen) macrophages infected with H37Ra [Fig. 1D]. The increase in IL-6 was observed in a dose dependent manner. Further, these results were corroborated by *IL-6* gene expression by RT-qPCR [Fig. 1E]. Additionally, we confirmed these results by observing a significant (p < 0.01) increase in the level of IL-6 and IFN-γ by IFG stimulated H37Ra infected macrophages isolated from human PBMCs [Fig. 1F,G]. Infergen is reported to promote Th1 polarization^32^. No noticeable change was seen in *IL-1β, TGF-β, IL-10* and *TNF-α* genes expression [Fig. S2A–D].

### IFG upregulates the expression of CD80, CD86 and HLA-DR

**Figure 2:**
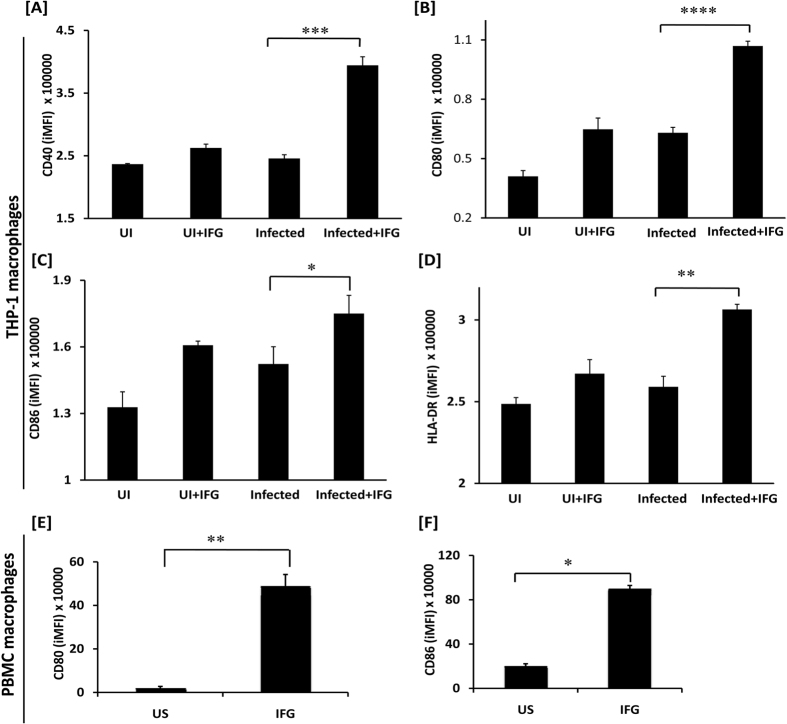
IFG upregulates the expression of CD40, CD80, CD86, and HLA-DR on the *Mtb* infected macrophages. [**A–D**] Macrophages; [**E,F**] Human PBMCs were infected with *Mtb* for 4 h and subsequently stimulated with IFG (64 ng/ml) for 24 h. Later, expression of CD40, CD80, CD86, and HLA-DR was evaluated by flow cytometry on the [**A–D**] macrophages; [**E,F**] CD11b gated PBMCs macrophages. The flow cytometry data (iMFI) represented through bar diagrams as mean ± SEM are representative of two independent experiments. UI: uninfected macrophages; UI + IFG: uninfected macrophages stimulated with IFG; US: *Mtb* infected macrophages; Infected + IFG: *Mtb* infected and IFG stimulated macrophages. *p ≤ 0.0285, **p < 0.003, ***p < 0.0004, ****p < 0.0001.

It has been reported that signaling through IFN-α/β can activate macrophages and other cells of the immune system during viral infection^31^,^32^,^33^,^34^,^35^. Further, MHC and costimulatory molecules expressed on the surface of the macrophages are crucial for the optimum activation of T cells. It was noticed that *Mtb*-infected IFG stimulated THP-1 macrophages (*Mtb*-Mφ-IFG) exhibited substantially (p < 0.02) higher expression of costimulatory molecules CD40 (p < 0.0004), CD80 (p < 0.0001), CD86 (p < 0.02) and MHC-II (HLA-DR) (p < 0.003) [Fig. 2A–D and Fig. S3A–D]. These results were substantiated using CD11b^+^ human macrophages. We observed that IFG induces significant upregulation of CD80 (p < 0.01), CD86 (p < 0.05), compared to unstimulated controls [Fig. 2E,F and Fig. S3E,F]. Thus, our data suggest that IFG not only augments the activation and maturation of *Mtb* infected Mφ differentiated from THP-1, but also macrophages isolated from human peripheral blood.

We further checked the activation status of other immune cells like B cells and T cells. As compared to untreated B cells, IFG treated B cells displayed a higher expression of CD80, CD86 and HLA-DR, [Fig. S4A–F]. We also noted significant (p < 0.01) upregulation of the activation marker CD25 on CD8 T cells treated with IFG [Fig. S5A]. Moreover, IFG incubated CD8 T cells showed substantial improvement (p < 0.01) in their ability to lyse the target cells [Fig. S5B]. Furthermore, IFG induced significant (p < 0.05) proliferation of PHA elicited human PBMCs [Fig. S5C]. These results designate that IFG not only stimulates the macrophages, but also B cells and CD8 T cells.

### IFG activated macrophages show enhancement in antigen uptake

**Figure 3:**
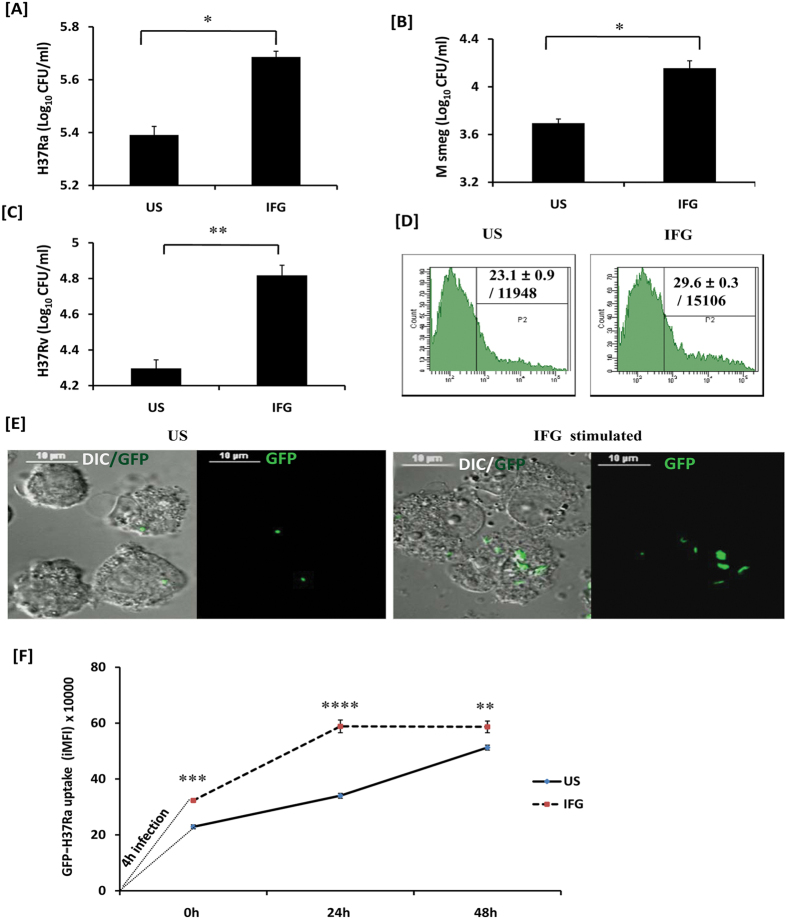
Signaling through IFG enhances phagocytosis. Macrophages stimulated with IFG for 24 h were infected with [**A**] H37Ra; [**B**] *M. smegmatis*; [**C**] H37Rv; [**D,E**] GFP-H37Ra. After 4 h, cells were lysed and bacterial uptake was monitored by [**A–C**] CFUs. [**D**] Flow cytometry; [**E**] confocal microscopy. [**A–C**] The data (mean ± SEM) shown as bar diagrams indicate bacterial uptake. [**D**] The data in the inset of histograms shown as % cells/MFI denote bacterial uptake. [**F**] The macrophages stimulated with IFG for 24 h and thereafter infected with GFP-H37Ra for 4 h. Time-point kinetics of bacterial uptake by macrophages was measured by flow cytometry. The flow cytometry data (iMFI) represented through the line diagrams as mean ± SEM are representative of two independent experiments. IFG: infected macrophages stimulated with Infergen; US: infected macrophages (not stimulated with IFG); DIC: differential interference contrast. Image magnification: 60x. Data are representative of two independent experiments. *p < 0.05, **p < 0.003, ***p < 0.0004, ****p < 0.0001.

Macrophages are extremely efficient APCs responsible for phagocytosis. Therefore, we were curious to check whether IFG stimulated macrophages acquire an enhanced capacity to phagocytose *Mtb*. Consequently, we stimulated macrophages with IFG and then infected them with *Mtb*. The extracellular bacteria were removed by extensive washings and subsequently treated with amikacin. Later, the cells were lysed and bacterial growth was enumerated by CFU plating. Remarkably, IFG significantly (p < 0.05) improved the ability of macrophages to phagocytose H37Ra, compared to unstimulated control [Fig. 3A]. These results were further supported by increased uptake of other mycobacterial strains *M. smegmatis* [Fig. 3B] and *Mtb-*H37Rv [Fig. 3C]. Furthermore, our flow cytometry and confocal microscopy experiments substantiated higher uptake of GFP^+^-H37Ra by IFG stimulated Mφ [Fig. 3D,E and Fig. S6A,B]. The Z-stack [Fig. S6C] and live imaging videos [Fig. S6D, video 1, 2] also confirmed the presence of GFP^+^-*Mtb* inside the macrophages. In addition, we validated the higher uptake capacity of GFP^+^-H37Ra by IFG stimulated Mφ through time point kinetics assay by flow cytometry [Fig. 3F]. Thus, these results signify the potential role of IFG in enhancing the phagocytosis by macrophages.

### Induction of iNOS expression by IFG is necessary for autophagy

**Figure 4:**
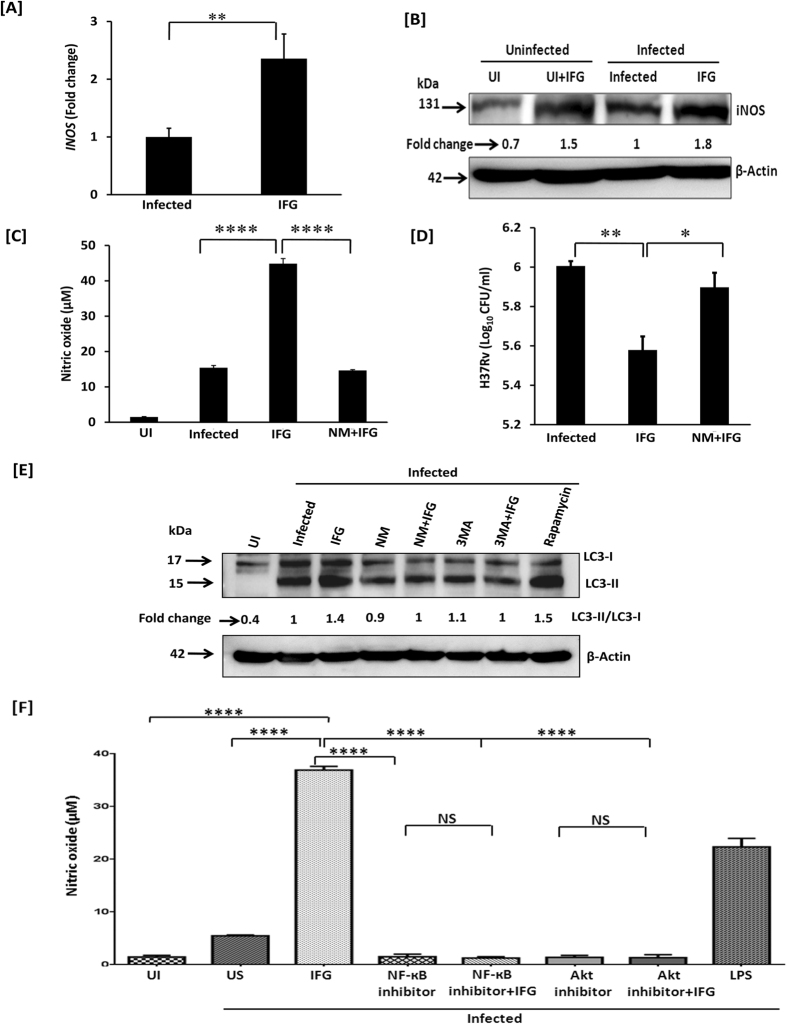
IFG restricts the intracellular survival of *Mtb* through the induction of iNOS. Macrophages were infected with *Mtb* for 4 h. Later, cells were stimulated with IFG (64 ng/ml) for 24 h and monitored for the expression of [**A**] *INOS* gene by RT-qPCR; [**B**] iNOS by Western blotting and values [fold change] are shown through densitometry analysis. [**C,D**] The specificity of NO production and its role in *Mtb* killing is proven by NO inhibitor NM. The estimation of [**C**] NO was done by Griess method and [**D**] *Mtb* killing by CFUs. The impact of autophagy in the inhibition of the growth of *Mtb* was ascertained by the [**E**] conversion of LC3-I to LC3-II by Western blotting and densitometry values are shown as fold change. Actin was used as a loading control. [**F**]. The secretion of NO was inhibited by treating the cells with the inhibitors of Akt and NF-κB. LPS was used as a positive control. The specificity of autophagy was ascertained by blocking the conversion of LC3-1 into LC3-II by its inhibitors 3MA and NM. Rapamycin is used as a positive control of autophagy. The fold change was calculated considering the value of infected controls as 1. The data are representative of 2–4 independent experiments. UI: uninfected macrophages; Infected: infected macrophages and unstimulated (no IFG); IFG: *Mtb* infected macrophages and IFG stimulated, NM: N-monomethyl-L-arginine, 3MA: 3 methyl adenine. *p < 0.05, **p < 0.01, ****p < 0.0001.

We next monitored the mechanism of action operating in IFG stimulated cells in restricting *Mtb* survival. Intriguingly, we observed that inhibition in the growth of *Mtb* by IFG stimulated cells was operating through NO production, as evidenced by an increase in the expression of *INOS* by RT-qPCR [Fig. 4A], as well as through expression of iNOS in the cytosol by Western blotting experiments [Fig. 4B]. Further, *Mtb* infected Mφ on stimulation with IFG produced remarkably more nitric oxide (NO), as compared to either unstimulated or infected control macrophages [Fig. 4C]. Nitric oxide is an established indicator of bactericidal activity^36^. We substantiated the specificity of the induction of iNOS by treating *Mtb* infected macrophages with iNOS inhibitor [(N-monomethyl-L-arginine), NM]. Interestingly, we observed significant decline in the NO production (p < 0.0001) and increase in the bacterial CFUs (p < 0.05) upon treatment of *Mtb*-MφIFG with NM [Fig. 4C,D]. We also showed that there was downregulation in the autophagy marker LC3, when iNOS was inhibited by NM [Fig. 4E]. Thus, establishing that iNOS activity is required for autophagy. Furthermore, autophagy inhibitor 3 methyl adenine (3MA) was used to unambiguously establish the induction of autophagy in *Mtb* infected Mφ treated with IFG [Fig. 4E]. Rapamycin was used as a positive control. Moreover, blocking of Akt-PI3K and NF-кB pathways by their respective inhibitors, substantially reduced the secretion of NO [Fig. 4F].

**Figure 5:**
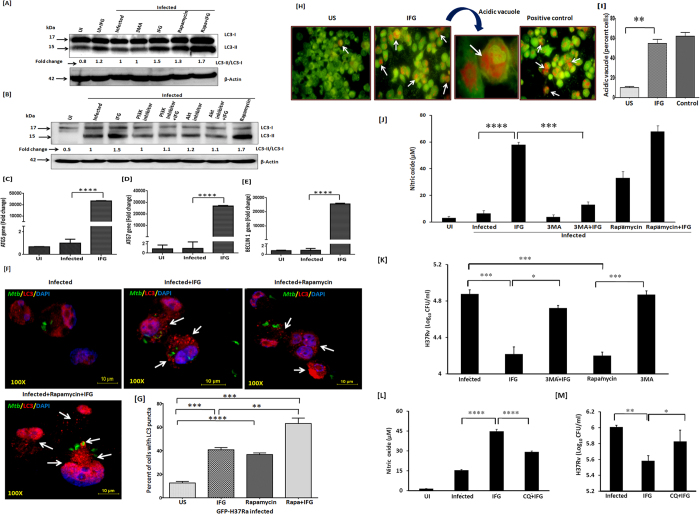
The mechanism involved in controlling the growth of *Mtb* by IFG is through autophagy. Macrophages were infected with *Mtb* (H37Rv) for 4 h. Later, cells were stimulated with IFG (64 ng/ml) for 24 h and monitored for [**A**] the augmentation in the IFG induced autophagy by rapamycin through conversion of LC3-I to LC3-II by Western blotting. The densitometry values are shown as fold change. [**B**] The specificity of autophagy was determined by blocking Akt-PI3K pathway by their respective inhibitors. Rapamycin is used as a positive control of autophagy and actin as a loading control. The fold change was calculated considering the value of infected controls as 1. [**C**–**E**] Upregulation in the expression of autophagy markers [**C**] ATG5; [**D**] ATG7; [**E**] BECLIN 1 was done by RT-qPCR and the results are represented as relative expression (fold change). The fold change was calculated considering the value of uninfected controls as 1. [**F,G**] The induction and over expression of autophagy influx by LC3 puncta formation through confocal microscopy after IFG+rapamycin treatment in GFP-H37Ra infected macrophages. The cells were infected with GFP-H37Ra [green] and stained for the expression of LC3 with anti-LC3 Abs [red], along with nucleus with DAPI dye [blue]. The frequency of puncta formation was enumerated using 4–5 different microscopic fields and the percent of cells with LC3 puncta [mean ± SEM] are presented as bar diagram. [**H,I**] Acidic vacuole formation was performed by acridine orange staining and fluorescence microscopy. The data [mean ± SEM] displayed as percent positive vacuole forming cells [orange] are from five different microscopic fields. The specificity of autophagy was determined by blocking its function by autophagy inhibitors [**J,K**] 3MA and [**L,M**] CQ; as observed by the change in the yield of NO and survival of *Mtb*. Rapamycin was used as a positive control. The data are denoted as mean ± SEM for NO [μM] and H37RV [CFU/ml]. The results are representative of 2–4 independent experiments. UI: uninfected, US: infected, but not stimulated with IFG, IFG: *Mtb* infected and IFG stimulated, NM: N-monomethyl-L-arginine, 3MA: 3 methyl adenine, CQ: Chloroquine. *p < 0.05, **p < 0.01, ***p < 0.001, ****p < 0.0001.

Autophagy plays a fundamental role in inhibiting the intracellular survival of *Mtb*^37^. Interestingly, IFG stimulation exhibited higher conversion of LC3I to LC3II, a hallmark of autophagy. We observed further amplification of autophagy by rapamycin treatment of the infected macrophages stimulated with IFG [Fig. 5A]. Thus corroborating the IFG induced autophagy. Additionally, blocking of Akt and PI3K pathways by their respective inhibitors inhibited the induction of autophagy [Fig. 5B]. Furthermore, augmentation (p < 0.0001) of the autophagy genes ATG5, ATG7 and BECLIN1, categorically established the involvement of autophagy [Fig. 5C–E]. Finally, we verified this finding by observing the over expression of autophagy influx by LC3 puncta formation in GFP-H37Ra infected macrophages through confocal microscopy after IFG and rapamycin treatment [Fig. 5F,G]. We observed that virulent and avirulent mycobacteria have similar responses with respect to autophagy. Interestingly, we also observed that IFG itself has an ability to induce autophagy influx in macrophages (Fig. S7A,B). Likewise, the acidic vacuoles were stained with acridine orange dye to show the induction of autophagy [Fig. 5H,I]. We also monitored autophagy by treating cells (infected and IFG stimulated) with rapamycin and correlated this with NO production and CFU burden [Fig. 5J,K]. Autophagy specific inhibitors 3MA and chloroquine (CQ) were used to establish the role of autophagy in restricting the growth of *Mtb* by IFG stimulated Mφ. Substantial (p < 0.0001) reduction in the secretion of NO and increase in the survival of *Mtb* was noticed [Fig. 5L,M]. Thus, these results signify that the function of iNOS and autophagy are interlinked with each other and are possible mechanism operating in MφIFG in constraining the *Mtb* growth.

### IFG induces the phosphorylation of STAT-3 and activates Akt-PI3K pathway

**Figure 6:**
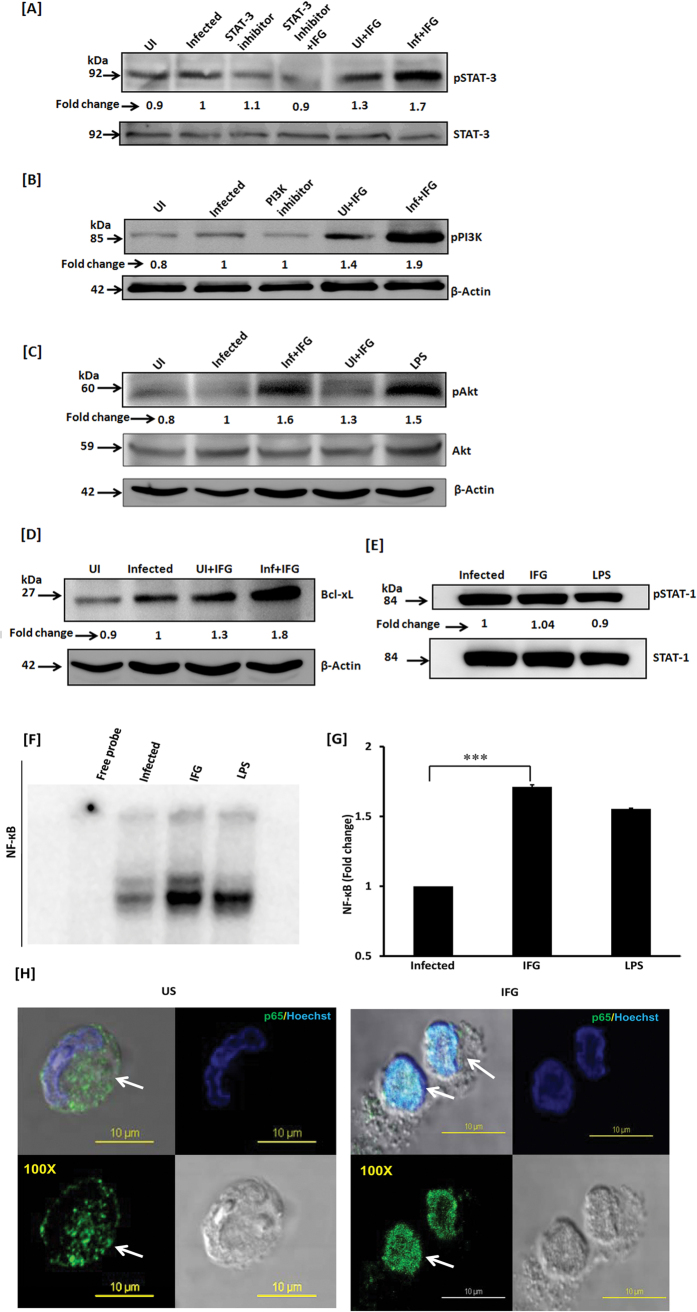
IFG signaling of the macrophages induces phosphorylation of STAT-3 through Akt-PI3K pathway and bolsters NF-κB nuclear translocation. *Mtb* infected macrophages were cultured with IFG. The cell lysate (cytosolic extract) was prepared and Western blotting was performed to monitor the expression of [**A**] pSTAT-3; [**B**] pPI3K; [**C**] pAkt; [**D**] Bcl-xL; [**E**] pSTAT-1. [**F,G**] NF-κB was detected using nuclear extract by EMSA and densitometry values are represented by bar diagram as a fold change (mean ± SEM). [**H**] The translocation of NF-κB from cytosol [US] to nucleus [IFG] was further validated by confocal microscopy using IFG stimulated *Mtb* infected macrophages. The green fluorescence indicates NF-κB marker p65 and blue signifies the nucleus staining with Hoechst dye (magnification: 100x). LPS is used as a positive control. US: infected macrophages [no IFG]; IFG: infected macrophages treated with IFG. Data are representative of two independent experiments. ***p ≤ 0.0002.

It has been well established that IFN-α/β interacts with its receptor IFNAR and activates tyrosine kinase Jak-1 along with Tyk-2^38^,^39^. Jak-1 stimulates and phosphorylates STAT-1/STAT-3. As a result, Akt is activated through STAT-3 along with PI3K and translocates into the nucleus. Further, it helps in delivering survival signal through the activation of NF-κB. It has been reported that like IFN-α/β, IFG exhibits affinity for IFNAR and activates tyrosine kinase Jak-1 along with Tyk-2. Therefore, we investigated the mechanism involved in IFG-mediated signalling events responsible for activating macrophages to restrict the intracellular growth of *Mtb. Mtb* infected macrophages were stimulated with IFG and phosphorylation of STAT-3, PI3K and Akt molecules were monitored by Western blotting in the cytosolic extract. Noticeable augmentation in the phosphorylation of STAT-3, PI3K and Akt was observed in the MφIFG [Fig. 6A–C]. The decrease in the phosphorylation of STAT-3 and PI3K was checked in Mφ-IFG by treating with their respective inhibitors [Fig. 6A,B]. Further, augmentation in the anti-apoptotic molecule, Bcl-xL was noticed [Fig. 6D]. Bcl-xL plays a vital role in providing a survival signal to the cells. No change was seen in the case of STAT-1 [Fig. 6E].

### IFG signaling augments the translocation of NF-κB into the nucleus

**Figure 7:**
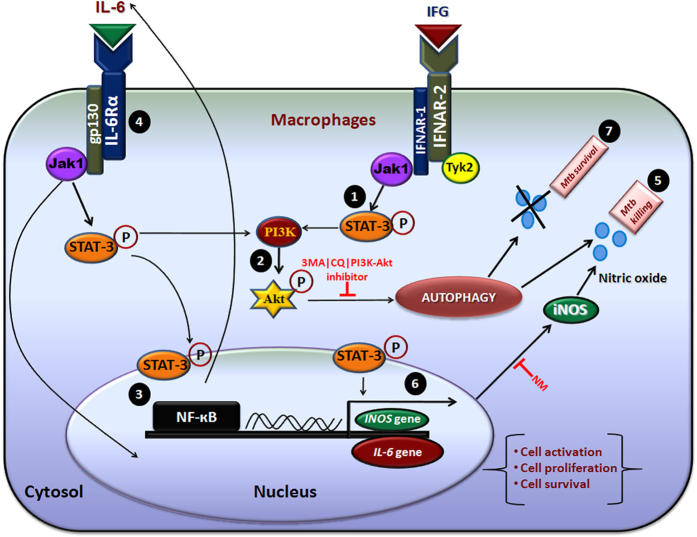
Proposed model of signaling pathway induced by IFG in the *Mtb* infected macrophages. It has been reported that IFG binds to IFNAR-2 and activates the tyrosine kinase Jak-1 and Tyk-2. Jak-1 induces and stimulates the phosphorylation of STAT-3 [pSTAT-3]^38^,^39^. We investigated the role of IFG mediated signaling mechanism in activating macrophages to restrict the intracellular growth of *Mtb*. **[1]** The phosphorylation of STAT-3 is induced by Jak-1. **[2]** Subsequently, Akt is activated through STAT-3 along with PI3K. **[3,4]** This signaling translocates NF-κB into the nucleus and upregulates the IL-6 secretion, which receives a feed-back mechanism through pSTAT-3, Akt and therefore induces autophagy. Thus, ensuring the activation and survival of the *Mtb* infected macrophages. **[5]** The induction of autophagy in the *Mtb* infected macrophages directly involved in the killing of *Mtb.* **[6]** We propose that pSTAT-3 binds to the promoter sequence of *INOS* gene to produce ions in the cytosol that generates NO, a potent antimicrobial agent that may ultimately restrict the growth of *Mtb*. The data were validated by blocking the Akt-PI3K, iNOS and autophagy with their respective inhibitors to establish the role of autophagy in restricting the growth of *Mtb* by IFG stimulated macrophages. **[7]** Blocking of the Akt-PI3K, iNOS (NM), autophagy (3MA|CQ) with their specific inhibitors and therefore substantial reduction in the secretion of NO and autophagy, as well as increase in the survival of *Mtb* signifies that autophagy is directly involved in the killing of *Mtb*. Signifying that the function of iNOS and autophagy are interlinked. Overall, this mechanism illustrates that IFG plays a key role in controlling the survival of *Mtb* inside macrophages.

NF-κB is responsible for cellular growth, inflammatory and immune responses. We observed a significant (p ≤ 0.0002) increase in the activation of NF-κB in H37Ra-Mφ-IFG [Fig. 6F,G]. Further, experiments were performed that confirmed the translocation of NF-κB by confocal microscopy in IFG stimulated cells [Fig. 6H]. This observation substantiated EMSA data [Fig. 6F]. Overall, this finding signifies that induction of autophagy in IFG stimulated macrophages operates through NF-κB, STAT-3 and Akt-PI3K pathways [Fig. 7].

## Discussion

There is an urgent need and a challenge to discover new drugs to treat TB because the disease continues to kill millions of people annually. Further, despite of the availability of potent drugs, the current regime remains quite complicated due to lengthy treatment, side effects and emergence of drug-resistant strains of *Mtb.*

Type 1 IFNs (IFN-α/β) are produced by macrophages, plasmacytoid DCs, fibroblasts and endothelial cells and play crucial effector function in viral infections^34^,^40^. Undeniably, previous references have indicated the efficiency of the IFNs therapy in treating yellow fever, chronic hepatitis B (HBV), hepatitis C (HCV) and ebola virus infections^41^,^42^. They augment the synthesis of several key antiviral mediators, including 2′-5′ oligoadenylate synthetase (2′-5′ A synthetase) and protein kinase R^43^,^44^. Further, type 1 IFNs upregulate CXCL10 expression in *Mtb* infected dendritic cells, which could be important to induce the homing and recruitment of cells to the granulomas^45^.

Considering the importance of IFN-α/β in activating cells of the immune system and bolstering immunity, a synthetic interferon IFG-α2b, which is known as Infergen (IFG) was synthesized^46^,^47^,^48^. Infergen has been reported to upregulate MHC-I expression and enhance the presentation of viral peptides by APCs. As a result, it enhances the activation and cytolytic activity of CD8^+^ cytotoxic T lymphocytes (CTLs)^49^,^50^,^51^. Further, like IFN-α/β, IFG exhibits an optimized affinity for IFNAR and augments the survival and activation of neutrophils, macrophages, DCs, NK cells, B cells and CD8 T cells^31^,^32^,^33^,^34^,^35^,^52^. Furthermore, it is reported that IFG activates various cell types to endorse viral clearance by promoting apoptosis of infected cells^34^,^52^. Unfortunately, nothing has been reported regarding the role of IFG against *Mtb*.

In the past, regular efforts have been made to investigate the impact of new molecules or delineate novel role of previously established molecules in the treatment of TB. Therefore, keeping in view the above-mentioned reports, we thought to evaluate the impact of IFG on the activation of macrophages and inhibition of the intracellular survival of *Mtb*. Following major findings have emerged from our study. IFG significantly i) inhibited the growth of *Mtb* in the macrophages; ii) augmented the secretion of IL-6 and IFN-γ; iii) upregulated the expression of HLA-DR and costimulatory molecules CD80 and CD86; iv) enhanced phagocytic potential of macrophages; v) enhanced CD8 T cell mediated target lysis; vi) the mechanism elucidated in curbing the survival of *Mtb* was through the involvement of nitric oxide and autophagy; vii) signaling pathway induced by IFG was through phosphorylation of STAT-3, Akt and activation of NF-κB.

For the optimum activation of T cells, two signals are required. First is the occupancy of T-cell receptor (TCR) by MHC-peptide complex. Second signal is also APCs driven in the form of costimulatory molecules like CD80 and CD86. In the absence of second signal, T cells undergo anergy, i.e. a state of unresponsiveness. Upregulation of HLA-DR, CD80 and CD86 by IFG indicate its potential role in the optimal activation of T cells. Further, it will help in counteracting *Mtb*-mediated downregulation of costimulatory molecules and thereby avoiding T cell anergy^23^. IFG predominantly promotes the generation of Th1 cells and CD8 T cells^32^,^49^,^50^,^51^. Both these cells are responsible for cell mediated immunity and play a cardinal role in protection against *Mtb*. CD8 T cells lyse the infected cells and release the intracellular *Mtb.* The IFN-γ secreted by Th1 cells activate macrophages and consequently they phagocytose and eliminate the bacterium. It was an exciting observation that IFG elicited macrophages showed enhanced phagocytosis and killing of *Mtb*. These results reveal a novel role of IFG against *Mtb.*

IFN-α/β interacts with IFNAR receptor and induces the activation of Jak-1 and Tyk-2. Jak-1 stimulates and phosphorylates STAT-3^38^,^39^. Consequently, Akt-PI3K is activated through STAT-3 and translocates into the nucleus. Further, Akt-PI3K-STAT-3 signaling helps in delivering survival signal through the activation of NF-κB^38^,^39^. Like IFN-α/β, IFG [(IFN)-α2b] also exhibits a specific affinity for IFNAR and activates tyrosine kinase Jak-1 along with Tyk-2^38^,^39^,^52^,^53^. It is also important to mention that signaling through IL-6 also follows the gp130/Jak/STAT pathway^54^. We investigated the signaling mechanism mediated by IFG in activating macrophages to restrict the intracellular survival of *Mtb*. We observed that *Mtb* infected macrophages when activated with IFG showed release of IL-6, but no noticeable change was seen in *IL-1β, TGF-β, IL-10, TNF-α* genes except *IL-6* gene expression and subsequent phosphorylation of STAT-3 and Akt but not STAT-1. This indicates that IL-6 pathway is involved in IFG signaling. Phosphorylation of STAT-3 and Akt-PI3K delivers a survival signal to macrophages through upregulation of Bcl-xL and activation of NF-κB, thereby ensuring cell survival, activation and induction of autophagy. We speculate that STAT-3 binds to a promoter sequence of *INOS* gene, enhances the synthesis of iNOS and therefore production of NO that ultimately leads to the killing of *Mtb* [Fig. 7]. The data were validated by blocking the Akt-PI3K, iNOS and autophagy by their respective inhibitors to establish the role of autophagy in restricting the growth of *Mtb* by IFG stimulated macrophages. Blocking of Akt-PI3K, iNOS and autophagy, and substantial reduction in the secretion of NO and autophagy, as well as increase in the survival of *Mtb* signifies that autophagy is directly involved in the killing of *Mtb.* Further, the function of iNOS and autophagy are interlinked with each other. This provides the unambiguous evidence of a possible mechanism operating in constraining the *Mtb* growth. It has been reported that NO suppresses mTOR expression, which leads to autophagy^55^. Corroborating with this observation, we hypothesize that NO directly suppresses the expression of mTOR, as shown by the enhancement of NO release by mTOR inhibitor rapamycin (autophagy inducer), which led to the induction of autophagy in *Mtb* infected and IFG stimulated human macrophages. In essence, this study provides a novel immunomodulatory and therapeutic role of IFG against intracellular pathogen *Mtb*, which may have enough potential in future to treat TB patients.

## Materials and Methods

### Ethics statement

This study was permitted by the Institutional Biosafety and Ethical Committees of Institute of Microbial Technology, Chandigarh, India. All blood related work and experimental protocols were approved by the Institutional Biosafety and Ethical Committees of Institute of Microbial Technology, Chandigarh for project license IBSC8/IMT/10.7.12, and ethical guidelines for biomedical research on human subjects by Central Ethics Committee on Human Research (CECHR), ICMR-2000. Human blood was collected from healthy volunteers. The informed consent was obtained from all volunteers.

### Reagents

Human Infergen was a kind gift from Dr. Eleanor Fish, The Department of Immunology, University of Toronto, Toronto, Canada.

THP-1 monocytes (ATCC TIB 202) were differentiated into macrophages by PMA (phorbol-12-myristate-13-acetate) (Calbiochem, San Diego, CA) for 16 h. The cells were washed 3x and rested for next 16 h in RPMI-1640 (GIBCO Invitrogen Corporation, Grand Island, NY) medium supplemented with FBS (10%), L-glutamine (2 mM), HEPES (10 mM), sodium bicarbonate (2.2 mg/ml), penicillin (0.7 mg/ml), streptomycin (1 mg/ml) at 37 °C/5% CO_2_. Human PBMCs were activated with PHA (2 μg/ml) (phytohemagglutinin PHA-M, Sigma-Aldrich, St. Louis, MO).

All the chemicals and reagents were purchased from Sigma Aldrich (St. Louis, MO) or otherwise mentioned. Free or fluorochrome conjugated antibodies (Abs): CD8-FITC, CD40 FITC, CD80-PE and PerCPefluor710, CD19-FITC, CD86-PE, HLA-DR-FITC and PEcy7, CD11b-APC, HLA-DR-PerCP 5.5, CD25-APC-Cy7 and the reagents used for cytokine ELISA were purchased from BD Pharmagin (San Diego, CA) and eBioscience (San Diego, CA). Fetal bovine serum (FBS) was purchased from Biological Industries (Kibbutz Beit Haemek 25115, Israel) and GIBCO (Grand Island, NY). L-pyruvate, L-glutamine, streptomycin and penicillin were from Serva (Heidelberg, Germany). Tissue culture grade plastic-ware was purchased from BD Biosciences (Bedford, MA). Abs used in Western blotting against pAkt (Thr308) [C31E5E], Akt (Pan) [C67E7] and PI3 kinase p85 (19H8) [4257] were purchased from Cell Signaling Technology (Danvers, MA). Abs to pSTAT-3 (pY705) [612357], STAT-3 [610190], pSTAT-1 (pY701) [612132] and STAT-1 [610115] were procured from BD Biosciences (Bedford, MA) and iNOS [Ab3523] from Abcam (Cambridge, UK). Inhibitors against STAT-3 [S3I-201], PI3K [Ly294002] were bought from Sigma Aldrich (St. Louis, MO), and Akt [Akt inhibitor IV, 124011], iNOS [NM, 475886] and NF-кB [481408] from Calbiochem (Billerica, MA). The GFP^+^ *Mtb*-H37Ra was a kind gift from Dr. Pawan Gupta, CSIR-Institute of Microbial Technology, Chandigarh, India.

### Activation of THP-1 cells by IFG

THP-1 monocytes (3 × 10^5^/well) were differentiated in 24 well plate with PMA (25 ng/ml) for 16 h. The cells were washed and then rested for next 16 h. Later, cells were stimulated with different concentrations of IFG (0–64 ng/ml) for 24 h.

### T cell proliferation

PBMCs (1.5 × 10^5^ cells/well) isolated from human blood by ficoll-diatrizoate (Histopaque-1077, Sigma-Aldrich, St. Louis, MO) density gradient centrifugation method were stimulated with PHA (2 μg/ml) and cultured with different concentrations of IFG (0–64 ng/ml) for 48 h in 96 well U-bottom plate. Later, ^3^H-thymidine (0.5 mCi/well) (Perkin Elmer, MA) was incorporated into the cultures and incubated for additional 16 h. Subsequently, the culture plates were harvested on glass-fiber filter mats by a Tomtec Harvester-96 (Hamden, CT). The radioactivity incorporated was measured using β-scintillation counter (Wallac-1450 Microbeta Trilux, Perkin Elmer, Waltham, MA) and expressed as counts per minute (cpm).

### The activation of macrophages, B cells and T cells by IFG

PBMCs (2.5 × 10^5^/well) were stimulated with IFG (64 ng/ml) in the presence or absence of PHA for 48 h. Later, cells were harvested and stained with fluorochrome conjugated Abs to detect the activation of macrophages (CD11b^+^/CD80^+^/CD86^+^/HLA-DR^+^), B cells (CD19^+^/CD80^+^/CD86^+^/HLA-DR^+^) and CTLs (CD8^+^/CD25^+^) and their isotype matched control Abs for 30 min/4 °C. Usual procedures of incubation and washings were followed at each step. Finally, cells were fixed in paraformaldehyde (1%). The flow cytometry data were acquired using FACS Calibur and FACS Aria-II and analyzed by DIVA and Flowjo softwares (BD Biosciences, San Jose and Williamson Way, Ashland, OR).

### Mycobacterial uptake by IFG stimulated macrophages

THP-1 monocytes (3 × 10^5^/well) were differentiated with PMA (25 ng/ml) for 16 h, followed by resting for additional 16 h in 24 well plate. Later, macrophages were stimulated with IFG (64 ng/ml) for 24 h in RPMI-FBS-10% at 37 °C/5% CO_2_. After 24 h, IFG stimulated cells were infected with *Mtb* strains H37Rv or H37Ra at MOI (1:5) for 4 h (3 h for *M. smegmatis*). This was followed by incubation with amikacin (2 μg/ml) for 1 h to kill extracellular bacteria. After 1 h, cells were lysed with saponin (0.1%) and lysate (100 μl) was plated on 7H11 agar plates. Bacterial colonies were enumerated on 21d. All subsequent experiments were performed with strain H37Rv (*Mtb*) or otherwise indicated.

### Phagocytosis of GFP^+^ *Mtb* by IFG stimulated macrophages by confocal microscopy

Macrophages (3 × 10^5^/well) were stimulated as mentioned above with IFG for 24 h. Later, cells were infected with GFP^+^ *Mtb* (H37Ra) [MOI:1:10] for 4 h. The amikacin (2 μ g/ml) treatment was given for 1 h to kill the extracellular bacteria, followed by extensive washing with cold PBS. Subsequently, cells were fixed with paraformaldehyde (1%) for 10 min. These cells were washed and placed in poly-L lysine pre-coated cover slips and imaged using Nikon A1 confocal laser microscope (Nikon, Tokyo, Japan). Z-stacks and extent of GFP^+^ *Mtb* internalization were monitored among experimental and control samples. The analysis of the intensity of surface plots was performed using Nikon NIS-AR 4.1 image analysis software (Nikon, Melville, NY). The confocal results were further validated by flow cytometry and analysis was done by FACS Suite software (BD Biosciences, San Jose).

### Intracellular killing of *Mtb* by IFG stimulated macrophages

Macrophages were infected with *Mtb* (H37Rv or H37Ra) at MOI (1:10) for 4 h in 24 w plates, followed by extensive washings with PBS (1X) to remove extracellular bacteria. Later, cells were cultured in antibiotic free RPMI + FBS-10% along with IFG (0–64 ng/ml) for 24 h. Amikacin (2 μg/ml) was added to the cultures to kill the extracellular *Mtb*. After 24 h, cells were lysed with saponin (0.1%) and lysate (100 μl) was plated on 7H11 agar plate. Colonies were enumerated on 21d.

### Cell culture and estimation of cytokines

THP-1 macrophages (2.5 × 10^5^/w) and macrophages (2.5 × 10^5^/w) isolated from the human PBMCs by adhering on tissue culture grade petri plates for 4–6 h were cultured with IFG (0–64 ng/ml) in 48 w plates for 48 h. The control cells were cultured in medium alone. The supernatants were collected after 24 h and IL-6 and IFN-γ were estimated. Briefly, 96 w plates were coated O.N/4 °C with Abs to human IL-6 (2 μg/ml) and IFN-γ (2 μg/ml) in phosphate buffer (pH 9.2, 0.01M). The unbound sites were blocked with BSA (1%) in PBS (1X) for 3 h/RT. Subsequently, respective recombinant cytokines as standard, and culture supernatants (50 μl volume) were added and incubated ON/4 °C. Later, biotinylated anti-human IL-6 (2 μg/ml) and IFN-γ (2 μg/ml) Abs diluted (1:1) in PBS-Tween buffer were added into the plates and incubated for 2 h/RT. Next, avidin-HRP (1:10,000) was added and incubated at 37 °C/45 min. The regular procedures of washing and incubation were followed at each step. The color was developed using H_2_O_2_-OPD substrate-chromogen. The reaction was stopped by H_2_SO_4_ (7%) after 20 min. The plates were read at 492 nm. The results expressed as pg/ml of secreted cytokines were calculated using serial log_2_ dilutions of standard curve drawn with rIL-6 and rIFN-γ.

### Viability of IFG treated macrophages

Macrophages were stimulated with IFG (0–64 ng/ml) for 24 h at 37 °C in RPMI-FBS-10% (500 μl) in 48 w plates. The stimulated cells were harvested, washed and resuspended in PBS and stained with 5 μl/tube annexin V and 2.5 μl/tube of propidium iodide (50 mg/ml). The cells were incubated in the dark for 15 min at RT. Later, PBS (200 μl) was added to the cells and acquired immediately using BD FACS Calibur flow cytometer and analysis was done by DIVA software.

### Real time PCR for the quantification of *IL-6, ATG5, ATG7, BECLIN-1, INOS, IL-1β, IL-10, TGF-β and TNF-α*

Total RNA was isolated using trizol reagent from IFG (64 ng/ml) stimulated macrophages for 6 h, according to the manufacturer’s instruction (Invitrogen, Carisbad, CA). Briefly, RNA was quantified with the help of NanoDrop spectrophotometer. A260/A280 ratio of all the samples was in the range of 1.90 to 2.00. DNA contamination from RNA samples was removed by amplification grade DNase. RNA samples (3 μg) were incubated with DNase (3U) for 15 min in the reaction buffer. Later, DNase activity was terminated by adding stop solution. Further, the samples were heated at 70 °C/10 min to inactivate DNase activity. Analysis was done by comparative Ct method, whereas Ct values were normalized against house-keeping control actin. The results are represented as relative expression (fold change). The fold change was calculated considering the value of uninfected/unstimulated controls as 1. RT-qPCR and data analysis were done by Step One Plus RT-qPCR system (Applied Biosystems, Chromos, Singapore). The RT-qPCR primer sequences are as follows:

***β-ACTIN*** **primer:** β-ACTIN-F 5′-AGAGGGAAATCGTGCGTGAC-3′

        β-ACTIN-R 5′-CAATAGTGATGACCTGGCCGT-3′

***IL-6*** **gene primer:** CIL-6-H R 5′-CAGGGGTGGTTATTGCATCT-3′

        CIL-6-H R 5′-CAGGGGTGGTTATTGCATCT-3′

***ATG5*** **gene primer:** ATG5-Fwd 5′-AGAAGCTGTTTCGTCCTGTGG-3′

        ATG5-Rev 5′-AGGTGTTTCCAACATTGGCTC-3′

***ATG7*** **gene primer:** ATG7-Fwd 5′-AATGTCTGCTGCTTGGAGCC -3′

        ATG7-Rev 5′-ACCCCCTAGGCAATCTTCAAA-3′

***BECLIN-1*** **gene primer:** BECLIN1-Fwd 5′-ACCTCAGCCGAAGACTGAAG-3′

        BECLIN1-Rev 5′-AACAGCGTTTGTAGTTCTGACA -3′

***INOS*** **gene primer:** EiNOS-H F 5′-ACAAGCCTACCCCTCCAGA-3′

        EiNOS-H R 5′-TCCCGTCAGTTGGTAGGTTC-3′

***IL-1β*** **gene primer:** hIL-1β-RT F 5′-GGACAAGCTGAGGAAGATGC-3′

        hIL-1β-RT R 5′-TCGTTATCCCATGTGTCGAA-3′

***TGF-β*** **gene primer:** CTGF-B1H F 5′-CTGGAAACCCACAACGAAA-3′

         CTGF-B1H R 5′-AACTTGAGCCTCAGCAGAC-3′

***TNF-α*** **gene primer:** hTNFα-RT F 5′-CACCATGAGCACTGAAAGCA-3′

         hTNFα-RT R 5′-CGAGAAGATGATCTGACTGCCT-3′

***IL-10*** **gene primer:** hIL10-RT F 5′-GAGAACAGCTGCACCCACT-3′

         hIL10-RT R 5′-CCCAGGTAACCCTTAAAGTCC-3′

### Target cell lysis by CD8 T cells

The target cells were prepared using PBMCs labelled with carboxyfluorescein succinimidyl ester (CFSE) (1 μM) for 8 min/37 °C in PBS (1X). The cells washed 3X with PBS-FCS 1% were γ-irradiated (3000R). Allogeneic PBMCs were isolated from second donor and T cells were purified and stimulated with IFG. Later, target and effector cells were cocultured (1:2 ratio). The percentage of CFSE-labeled PBMCs lysed by CD8 T cells were analyzed by flow cytometry. The formula was used to analyze specific lysis: ratio = (mean percentage of CFSE^hi^ cells/mean percentage of CFSE^lo^ cells), and the percentage of specific lysis = [1 − (ratio for CFSE^hi^ cells/ratio for CFSE^lo^ cells)] × 100.

### Nitric Oxide (NO) production

Macrophages were infected with *Mtb* for 4 h and stimulated with IFG (64 ng/ml) for 24 h. Later on, supernatants were collected and NO was estimated by Griess method^36^. Briefly, supernatants (50 μl) were incubated with an equal volume of Griess reagent along with sodium nitrite as standard for 5–10 min/RT. Later, absorbance was measured at 550 nm. Further, the involvement of NO in IFG signaling was authenticated by treating the cells with iNOS inhibitor (NM: N-monomethyl-L arginine) (20 μM), Akt inhibitor IV (20 μM) and NF-кB inhibitor (50 μM) for 1 h and stimulated with IFG for 24 h.

### Inhibition of iNOS expression by N-monomethyl-L arginine

*Mtb* infected Mφs were stimulated with IFG in the presence or absence of iNOS inhibitor (NM: N-monomethyl-L arginine) (20 μM) for 24 h. Later, cells were lysed and CFU plating was done to quantify the viable bacteria.

### Cell cultures and Western blotting

The *Mtb* infected macrophages were incubated with IFG (64 ng/ml). Further, to prove specifity, the cultures were treated with specific inhibitors for STAT-3 [S3I-201] (50 μM), PI-3K [Ly294002] (20 μM), Akt (20 μM), iNOS [NM] (20 μM) and autophagy [3MA] (20 mM) for 1 h and stimulated with IFG (64 ng/ml) at different time-points [16 h for iNOS, 4 h for LC3B, 15–30 min for STAT-3 | Akt | STAT-1 | Bcl-xL | PI3K] in 12 w plate. Cells stimulated with LPS (2 μg/ml) were used as a positive control. These cells were then harvested, washed, and lysed in lysis buffer (Cytoplasmic protein extraction buffer, phosphatase and protease inhibitor cocktail). Later, protein was estimated in the lysate and SDS-PAGE was performed. After transfer to PVDF membranes and subsequent blocking, the membranes were immunoblotted using Abs to phosphorylated and non-phosphorylated form of STAT-3, Akt, STAT-1 and PI3K. Blots were also incubated with Abs against LC3B, Bcl-xL, iNOS and loading control actin. Blots were developed using a chemiluminescence kit (Amersham Pharmacia Biotech, Buckinghamshire, UK) (ECL; Pharmacia-Amersham, Freiburg, Germany). Scanning of the blots was done by ImageQuant LAS 4000 (GE Healthcare, Freiburg, Germany). Image analysis was performed with MultiGuage image and ImageJ analysis softwares.

### Quantification of autophagy influx by confocal microscopy

The THP-1 macrophages were infected with GFP-H37Ra for 4 h and stimulated with IFG (64 ng/ml) and rapamycin [1 μM] for another 4 h. *Mtb*-Mφ-IFG were fixed on poly-L-lysine coated cover slips for 15 min. Later, cells were fixed with paraformaldehyde (2x) for 10 min followed by treatment with triton X100 (0.1%) for 3 min. The non-specific sites on cells were blocked with BSA (2%) for 2 h. This was followed by incubation with rabbit anti-human LC3 Ab for 2 h. Subsequently, cells were incubated with Alexa Fluor 633-anti-rabbit Ab for 1 h and followed by staining with DAPI dye. Regular washings were followed at each and every step. The cells were imaged through confocal microscopy. Rapamycin was used as a positive control. Number of puncta formation was counted manually from 4–5 different microscopic field. Percent of cells with LC3 puncta were presented in bar diagram.

### Demonstration of NF-κB by EMSA

Macrophages were cultured with IFG for 30 min. Later, the cells were harvested and nuclear extract was prepared for EMSA. Briefly, an equal amount of nuclear extract (3 μg) from each sample was incubated for 20 min/37 °C in water bath with [^32^P] end labeled duplex oligonucleotides containing binding site for NF-κB. The DNA–protein complexes were resolved by electrophoresis on a native PAGE-gel (7%). After electrophoresis, the native gel was dried and exposed to screen at RT for 6–10 h and scanned by phospho-imager (Fujifilm, Tokyo, Japan).

### The translocation of NF-κB by confocal microscopy

MφIFG were fixed on poly-L-lysine (0.1%) coated cover slips for 15 min. Later, cells were fixed with paraformaldehyde (2x) for 10 min followed by treatment with triton X100 (0.1%) for 3 min. The non-specific sites were blocked with BSA (2%) for 2 h. It was followed by incubation with mouse anti-human NF-κB p65 Ab for 2 h. Subsequently, cells were incubated with FITC-anti-mouse Ab for 1 h and followed by staining with Hoechst dye. Regular washings were followed at each step. The cells were imaged through Nikon A1 confocal laser microscope (Nikon, Tokyo, Japan) and analysis was performed using Nikon NIS-AR 4.1 image analysis software (Nikon, Melville, NY).

### Statistics

The data analysis was done by Student’s ‘t’ test, non-parametric Mann-Whitney two tailed and repeated measure ANOVA with post Student-Newman-Keuls multiple comparison test using Graph Pad InStat 3 and Graph Pad Prism 6 softwares.

## Additional Information

**How to cite this article**: Pahari, S. *et al*. Infergen Stimulated Macrophages Restrict *Mycobacterium tuberculosis* Growth by Autophagy and Release of Nitric Oxide. *Sci. Rep.* **6**, 39492; doi: 10.1038/srep39492 (2016).

**Publisher's note:** Springer Nature remains neutral with regard to jurisdictional claims in published maps and institutional affiliations.

## Supplementary Material

Supplementary Information

Supplementary Video 1

Supplementary Video 2
