# Supplementary material for: Infergen Stimulated Macrophages Restrict Mycobacterium tuberculosis Growth by Autophagy and Release of Nitric Oxide
Source: Sci Rep. 2016 Dec 21;6:39492. doi: 10.1038/srep39492 (PMC5175149; doi:10.1038/srep39492)
Supplement: Supplementary Information [file srep39492-s1.doc]

**Infergen Stimulated Macrophages Restrict *Mycobacterium tuberculosis* Growth by Autophagy and Release of Nitric Oxide**

**Susanta Pahari¹,², Nargis Khan¹, Mohammad Aqdas¹, Shikha Negi¹, Jagdeep Kaur²,**

**Javed N Agrewala¹***

¹ Immunology Laboratory, CSIR-Institute of Microbial Technology, Chandigarh-160036.

² Department of Biotechnology, Panjab University, Chandigarh-160014, India.

*Corresponding author: Javed N. Agrewala, CSIR-Institute of Microbial Technology,

Chandigarh-160036, India. E-mail: javed@imtech.res.in

**Supplementary figures**

**Figure S1. *IFG does not affect the cell viability.*** *Mtb* infected THP-1 macrophages were stimulated with IFG for 24h. Later, viability was assessed by PI/Annexin V staining. [A] Data shown as dot plots are percentage of PI/Annexin V positive cells and representative of two independent experiments. [B] THP-1 macrophages; [C] human PBMCs were stimulated with IFG for 24h. Later on, the cell viability was assessed by PI staining.

**Figure S2. *Macrophages stimulated with IFG show no change in the level of IL-1β*, *TGF-β*, *IL-10* and *TNF-α.*** Macrophages were incubated with IFG for 6h. RNA was isolated and the expression of [A] *IL-1β*; [B] *TGF-β*; [C] *IL-10;* [D] *TNF-α* was monitored by RT-qPCR. Data represented as mean±SD are of triplicate wells and two independent experiments. US: unstimulated cells (no IFG), NS: non-significant.

**Figure S3. *IFG upregulates the expression of CD40, CD80, CD86 and HLA-DR on the Mtb infected macrophages*.** [A-D] Macrophages and [E-F] human PBMCs were infected with H37Ra for 4h and subsequently stimulated with IFG for 24h. Later, expression of CD40, CD80, CD86, and HLA-DR was evaluated by flow cytometry on macrophages and CD11b gated PBMCs. The data shown in the inset are percent positive cells and representative of two independent experiments.


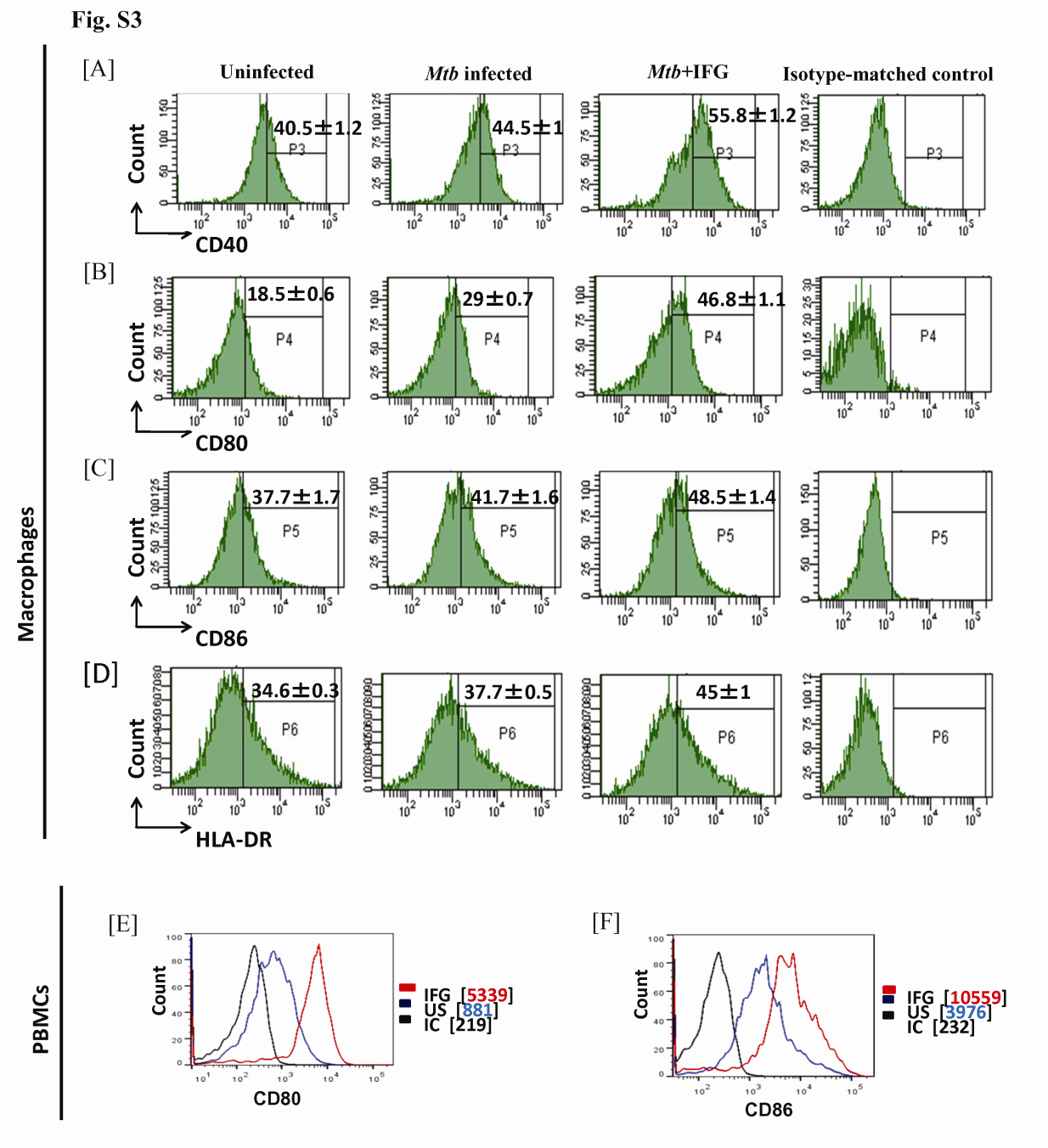


**Figure S4.  *IFG upregulates CD80, CD86, and HLA-DR on B cells*.** PBMCs were stimulated with IFG [64 ng/ml] along with PHA for 24h. Later, expression of [A, B] CD80; [C, D] CD86; [E, F] HLA-DR was evaluated on CD19^+^ B cells by flow cytometry. Data shown as mean±SEM are representative of two independent experiments. US: unstimulated. **p≤0.0046.

**
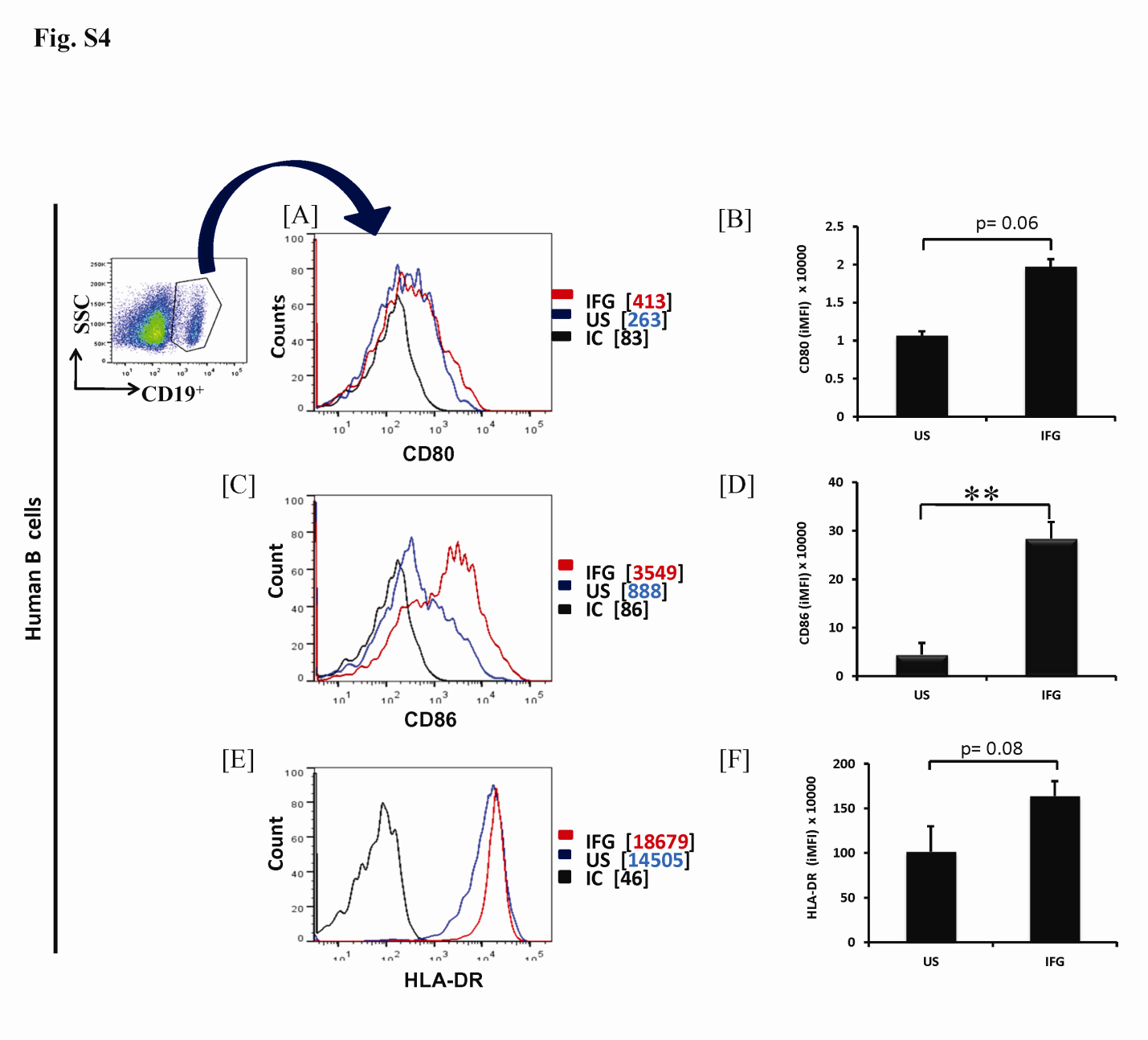
**

**Figure S5. *IFG activates CD8 T cells and enhances their ability to lyse targets.*** [A] PBMCs were cultured with PHA and IFG for 24h. The activation of CD25 was demonstrated by flow cytometry on CD8 gated T cells. The bar diagram signifies flow cytometry data (iMFI). [B] Purified human CD8 T cells stimulated with IFG for 24h were used as effector cells. CFSE-labeled and γ-irradiated allogeneic PBMCs were used as target cells. Both the cells were co-cultured [E:T, 1:2 ratio] for 4h. Target lysis was monitored by flow cytometry. The data are expressed as percent of target cells lysed by CD8 T cells. The results (mean±SEM) are from duplicate wells of two independent experiments. *p<0.05, **p≤ 0.0073. US: Unstimulated. [C] PHA stimulated PBMCs were incubated with IFG for 48h. Later, ^3^H-thymidine (0.5µCi/well) was incorporated into the cultures and cells were harvested. The radioactivity incorporated was measured by β-scintillation counting. Data shown are mean±SEM and representative of two independent experiments. SI: Stimulation index. *p≤0.0158.


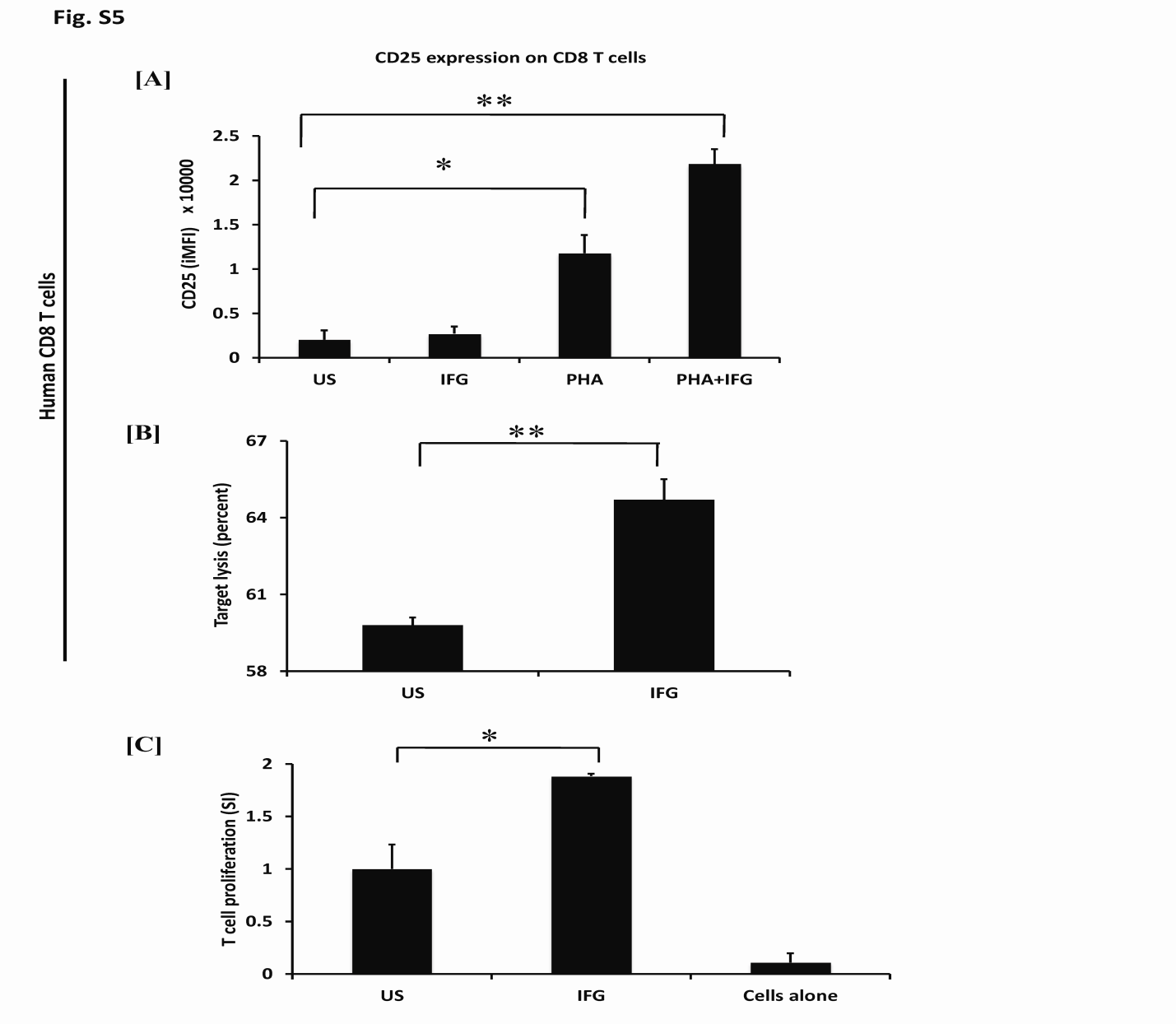


**Figure S6.** ***Signaling through IFG enhances phagocytosis.*** Macrophages stimulated with IFG for 24h were infected with [A-C] GFP-H37Ra. After 4h, cells were lysed and bacterial uptake was monitored by confocal microscopy. MFI illustrates bacterial uptake that is shown by [A] fluorescence intensity surface plots; [B] bar diagram. The unstimulated cells (no IFG) were taken as control. [B] The data shown by bar diagrams are mean±SEM and indicate bacterial uptake. US: unstimulated; FI: fluorescence intensity; DIC: differential interference contrast. Image magnification: 60x. [C] Z-stack images; [D] the confocal videos substantiate the results that IFG stimulated macrophages show augmented phagocytosis of GFP-H37Ra as compare to unstimulated control. Data are representative of two independent experiments. **p<0.003.


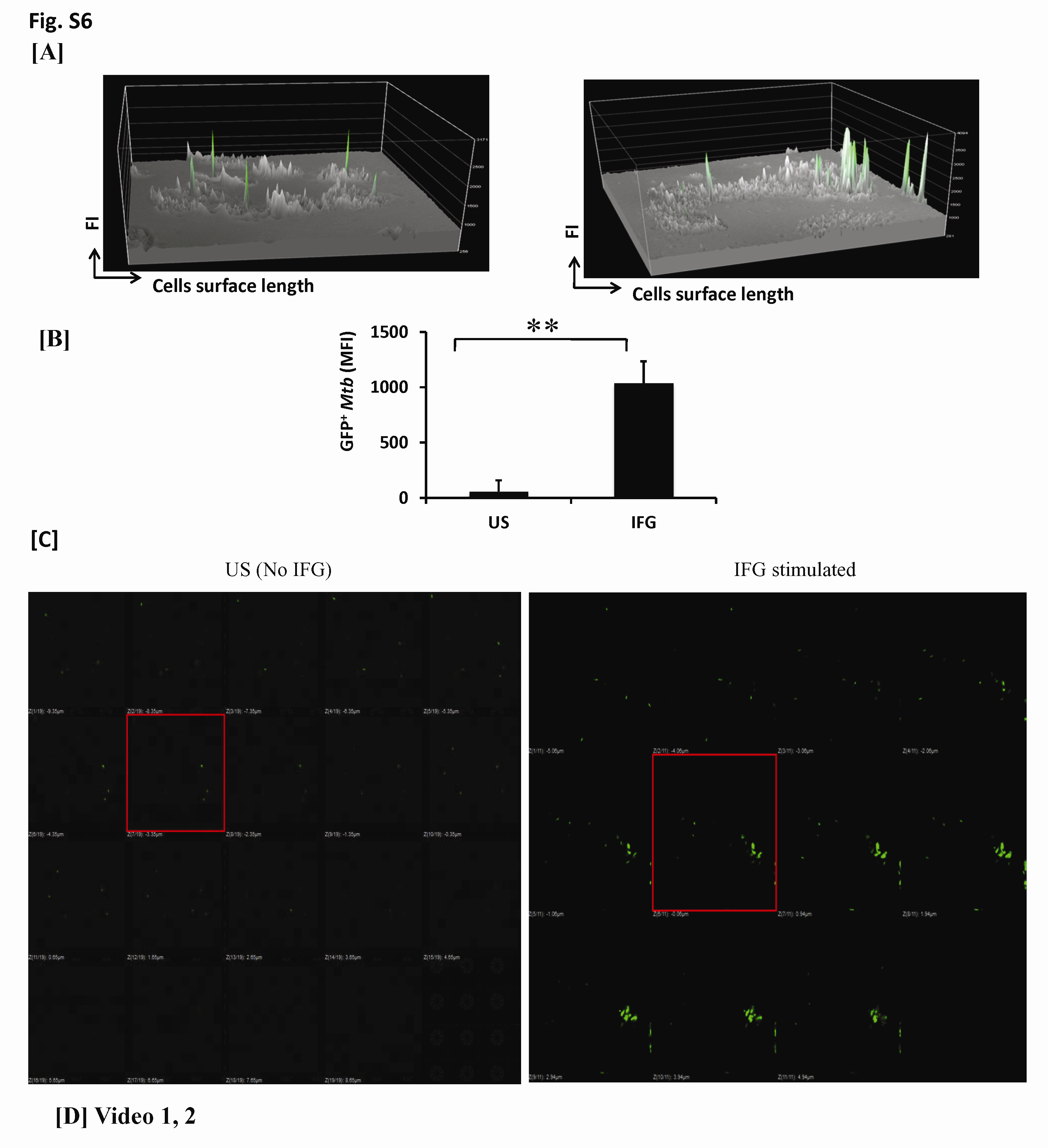


**Figure S7.** ***Signaling through IFG enhances autophagy.*** Macrophages were stimulated with IFG (64 ng/ml) for 4h. [A] The induction of autophagy influx by LC3 puncta formation through confocal microscopy after IFG treatment in THP-1 macrophages. The cells were stained for the expression of LC3 with anti-LC3 Abs [green], along with nucleus with Hoechst dye [blue]. The frequency of puncta formation was enumerated using 4-5 different microscopic fields. [B] The percent of cells with LC3 puncta [mean±SEM] are presented as bar diagram. **p<0.005.

**Video legends:**

THP-1 macrophages were stimulated with IFG (64ng/ml) for 24h and infected with GFP-H37Ra. After 4h, cells were washed and phagocytosis of GFP-H37Ra was monitored by confocal microscopy.

**Video 1 and 2.** The confocal videos show that IFG stimulated macrophages have augmented phagocytosis of GFP-H37Ra as compare to unstimulated control. Video 1: GFP-H37Ra phagocytosis by unstimulated macrophages. Video 2: GFP-H37Ra phagocytosis by IFG stimulated macrophages.
